# Supplementary material for: Vibrational exercise for Crohn’s to observe response (VECTOR): Protocol for a randomized controlled trial
Source: PLoS One. 2025 Mar 19;20(3):e0319685. doi: 10.1371/journal.pone.0319685 (PMC11922268; doi:10.1371/journal.pone.0319685)
Supplement: S1 File — (PDF) [file pone.0319685.s001.pdf]

Vibrational Exercise in Crohn’s disease to Observe Response; a randomised, controlled trial.
VECTOR
Study Protocol

Short title: VECTOR

Version number: 4

Funder: Crohns and Colitis Foundation, USA

Funder reference:

Sponsor(s): University of

Hertfordshire

Sponsor reference:

Clinical trial registration number (if applicable):

IRAS reference number (if applicable): 339311

REC reference (if applicable):

UH Protocol Number:

Table of Contents

General Information 5
Signature Page 6
Glossary of Abbreviations and Key Terminology 7
Trial/Study Summary 8
1. Introduction 10
2. Aims and Objectives 11
2.1. Trial/Study objectives 11
3. Methods 12
3.1. Study Design 12
VECTOR is a randomised controlled trial 12
3.2. Trial/Study Setting 12
3.3. Trial/Study Participants 12
4. Trial/Study Procedures 13
4.1. Trial/Study Setup 13
4.2. Recruitment 14
4.2.1. Participant Identification 14
4.3. Screening 14
4.3.1. Informed Consent 14
4.4. Allocation/Randomisation 14
4.4.1. Randomisation Procedures 14
4.5. Baseline 15
4.6. Intervention(s) 15
4.7. Follow up Procedures 16
4.8. End of Study 16
5. Safety 16
6. Data Collection, Management and Analysis 16
6.1. Data Collection 16
6.2. Data Management 17
6.3. Data Analysis 17
7. Ethical Considerations 18
8. Quality Assurance and Control 18
8.2. Risk Assessment 18
8.3. Monitoring 18

8.4. Study Oversight....18
8.4.3. Trial Management Group....18
9. Public and Patient Involvement....19
10. Protocol Compliance....19
11. Data Protection and Participant Confidentiality....19
12. Publication and Dissemination....20

General Information

This document provides details regarding the setting up of, conduct, analysis and dissemination of the Crohn's and Colitis Foundation funded trial/study (REF:339311, VECTOR).

The University of Hertfordshire will sponsor this trial/study. NHS sites with Gastroenterology clinics will be collaborators in this study. As such, a collaboration agreement will be signed by the parties, specifying responsibilities and financial arrangements.

Chief Investigator	Lindsay Bottoms
Academic Lead (if applicable)	
Trial/Study Co-ordinator/ Manager	Jo Brooks-Warburton
Sponsors	University of Hertfordshire
Study committees	Trial Management Group:<br>Dr Lindsay Bottoms<br>Dr Simon Anderson<br>Dr Amit Pujari<br>Dr Jonathan Sinclair<br>Dr Jo Brooks-Warburton<br>Dr Charlotte Lawson<br>Rachael Baker<br>Dr Matt Jewiss<br>CTSN representative<br>Dr Lauren Baker<br><br>Trial Steering Group (TBC)

Signature Page

The undersigned confirm that the following protocol has been agreed and accepted and that the Chief Investigator(s) agrees to conduct the trial/study in compliance with the approved protocol and will adhere to the principles outlined in the Good Clinical Practice (GCP) guidelines, the Sponsor's (and any other relevant) Standard Operating Procedures (SOPs), and other regulatory requirements as amended.

We agree to ensure that the confidential information contained in this document will not be used for any other purpose other than the evaluation or conduct of the investigation without the prior written consent of the Sponsor.

We also confirm that we will make the findings of the trial/study publicly available through publication or other dissemination tools without any unnecessary delay and that an honest, accurate and transparent account of the trial/study will be given; and that any discrepancies and serious breaches of GCP from the trial/ study as planned in this protocol will be explained.

Name:	Role: Chief Investigator	Signature:<br><br>Date:
Name:	Role: Sponsor	Signature:<br><br>Date:
Name:	Role: CTSN/CTU Director or Representative (if applicable)	Signature:<br><br>Date:
Name:	Role: Statistician (if applicable)	Signature:<br><br>Date:
Name:	Role: Health Economist (if applicable)	Signature:<br><br>Date:

Glossary of Abbreviations and Key Terminology

Organisations and personnel involved in the delivery of the study:
University of Hertfordshire, East and North Hertfordshire NHS Trust, Guys and St Thomas NHS Trust, Luton and Dunstable NHS Trust

Abbreviations and definitions:

AE	Adverse Event
AR	Adverse Reaction
ASR	Annual Safety Report
CI	Chief Investigator
CRF	Case Report Form
GAfREC	Governance Arrangements for NHS Research Ethics Committees
ICF	Informed Consent Form
Main REC	Main Research Ethics Committee
NHS R&D	National Health Service Research & Development
PI	Principal Investigator
QA	Quality Assurance
QC	Quality Control
Participant	An individual who takes part in a trial/study
RCT	Randomised Controlled Trial
REC	Research Ethics Committee
SAE	Serious Adverse Event
SDV	Source Document Verification
SOP	Standard Operating Procedure
SSA	Site Specific Assessment
TMG	Trial Management Group
TSC	Trial Steering Committee

Trial/Study Summary

Full title	Vibrational Exercise in Crohn's Disease to Observe Response, A Randomised Controlled Trial
Short title/Acronym	VECTOR
Protocol Version Number and Date	*V4 09/12/24*
Start Date	*September 2024*
End Date	*September 2027*
Trial/Study Duration	*36 Months*
Trial/Study Design	*Randomised Controlled Trial*
Sponsor/Co-sponsors	*University of Hertfordshire*
Chief Investigator(s)	*Lindsay Bottoms*
Funder	*Crohns and Colitis Foundation, USA*
REC Number	*339311*
Trial/Study Objective(s)	*1. To recruit 168 adult patients with mild to moderately active Crohn's disease to a fully powered randomised controlled trial, randomised 1:1 to a whole body vibration (WBV) programme with lifestyle education or to a control group with lifestyle education*<br>*2. To assess exercise capacity, health related quality of life, pain, fatigue, disease activity and inflammatory markers at baseline and after 6 weeks of either WBV or lifestyle education with a primary outcome of mean increase in 16 points on IBD-Q, indicating a significant improvement in quality of life*<br>*3. Secondary objectives include identifying the effect size*
Planned Sample Size	*168 patients*

Participants	*Inclusion criteria are:*<br>- *Is the patient aged between 18 and 65 years old (inclusive)?*<br>- *Has the patient had a clinical diagnosis of Crohn’s disease for at least 4 weeks before the screening visit?*<br>- *Does the patient have either mild or moderate Crohn’s disease based on a partial Harvey Bradshaw Index (5-16)?*<br>- *Does the patient have a faecal calprotectin <250 mcg/g recorded no greater than 4 weeks before the screening visit?*<br>- *Have the patient’s medications been stable (i.e., unchanged) for at least 4 weeks before the screening visit?*<br>- *Is the patient capable of giving written informed consent?*<br>- *Is the patient capable of completing the study questionnaires?*<br>- *Is the patient able to travel to the research sites for all assessment visits and exercise sessions?*<br><br>*Exclusion criteria:*<br>- *Does the patient have any absolute contraindications to exercise testing and training?*<br>- *Does the patient have any coexistent serious autoimmune disease?*<br>- *Does the patient have major surgery planned in the first 3 months following the anticipated baseline visit date?*<br>- *Is the patient pregnant?*<br>- *Is the patient female and planning pregnancy within the first 3 months following the anticipated baseline visit date?*<br>- *Does the patient think that they will be unable to tolerate the venepuncture requirements of the study?*<br>- *Does the patient lack sufficient venous access to meet the blood sampling requirements of the study?*<br>- *Is the patient participating in another clinical trial for with concurrent participation is deemed inappropriate?*<br>- *Is the patient unable to consent?*

Intervention	*After initial assessments (bloods for inflammatory markers, Chester step test, blood pressure, heart rate, faecal calprotectin, electromyelograph of the thighs), participants with be randomly allocated to either a 6 week whole body vibration course and lifestyle education programme or a control group with lifestyle education. The whole body vibration means the participant is supervised crouching (static squat) on a vibration plate for 60 seconds at a time, 6 times, with 60 second rests in between, three times per week.*<br>*Both groups will get the lifestyle education programme which will equate to 30 minutes per week with two sessions on healthy eating, 3 sessions on physical activity and 1 round up or smoking cessation education. Both groups will undertake an electromyelograph of their lower limbs (thigh muscles) at the beginning and end of the study. Both groups will undertake health related quality of life questionnaire (IBD-Q), HADS and IBD-Fatigue questionnaires, at the beginning and end of the study, as well as scoring their pain and level of physical activity daily one week prior and 1 week after the 6 weeks intervention, via REDCAP. The first 15 participants will be invited to participate in semi-structured interviews designed to gain feedback about how they felt the intervention went. Both groups will be asked to wear a chest strap and watch between the hours of 7am – 7pm, for three consecutive days following their first and last visit to the University of Hertfordshire. This is to assess heart rate variability.*
Comparison	*The comparator group will undertake the same as above, except without the WBV and the semi structured interviews.*
Follow up duration	*Data collection time points are visit 1 (pre starting WBV) , 6 weeks of WBV with data collection being EMGs, week 7 for bloods, stool, EMG and Questionnaires and week eight for the semi structured interviews. This will conclude the*
Outcomes	*1. Change in health related quality of life indices (IBD-Q questionnaire)*<br>*2. Change in perceived fatigue (using questionnaire IBD-F) and muscle fatigue (using EMGs)*<br>*3. Static or improvement in pain perception*<br>*4. Measurement of disease activity with HBI, inflammatory markers in blood samples and faecal calprotectin*<br>*5. Qualitative markers of tolerability of WBV as a therapy.*<br>*6. Change in heart rate variability*

1. Introduction

Crohn's disease is an incurable recurrent inflammatory bowel disease (IBD). Treatment is focused on managing the underlying inflammation or disease activity in the gastro intestinal tract, and thereby improving health related quality of life (HRQoL). However, disease activity is not the sole determinant of HRQoL, and multiple studies and reviews looking at interventions to improve all aspects of HRQoL, including dietary interventions, distance self-management and psychological therapy have all identified a need for interventions that are simple, safe, low cost and can improve several aspects of health and wellbeing. Exercise as an intervention is a candidate intervention.

Most research has focused on light to moderate aerobic exercise in IBD with a few studies investigating resistance type exercise. However, there appears to be no research study investigating whole body vibration (WBV) which has the potential to affect HRQoL by positively influencing inflammation, fatigue, bone mineral density, muscle strength, aerobic capacity, and pain. Being able to prescribe WBV exercise for IBD patients as an adjunct therapy could improve the patient’s HRQoL at a lower economic cost.
In the last decade vibration stimulation or so-called vibration therapy/exercise has become increasingly popular as an exercise technique in healthy individuals as well as for health conditions such as fibromyalgia and has been proposed as a relevant management technique for COVID19. Whole body vibration involves standing statically on an oscillating (i.e. vibrating) platform. Because the structure of the human body is not rigid, muscles and tendons act as springlike elements that store and release mechanical energy. This can result in increased muscle activity which can also increase heat production as well as increase blood flow.

Investigations using novel devices developed by one of the Co-Investigators have shown that in the upper limb muscles, vibration exercise superimposed on isometric contractions is able to generate significantly higher level of neuromuscular load leading to muscle activity in healthy individuals. Further, in the lower limbs we have shown that vibration superimposed on isometric contraction is able to induce significantly higher levels of neuromuscular activity compared to isometric contraction alone and that this increased neuromuscular activity is dependent on the level of muscle stretch, vibration frequency as well as amplitude. This demonstrates that static WBV exercise produces a greater stimulus than static exercise alone.

There are however barriers to exercise interventions - with time constraints being one of them. Research by Tew et al. (2019), has demonstrated that adults with IBD did not meet the recommended physical activity guidelines and that abdominal and joint pain was the greatest reason for limiting their physical activity followed by fatigue/tiredness and increased toilet urgency.

Regarding time constraints: WBV is a strong potential candidate for an exercise in intervention in this context as most research involving WBV have been of shorter durations 4-6 weeks compared to 10-12 weeks with aerobic exercise interventions. In addition, each individual training session tends to be of a much shorter duration; 10-15 minutes compared to 30 minutes of aerobic exercise.

In terms of pain management and treatment: it is hypothesised that WBV exercise could be utilised in a fashion similar to transcutaneous electrical nerve stimulation (TENS) in order to reduce the perception of pain. A systematic review and meta-analysis on the effects of WBV exercise on chronic musculoskeletal pain concluded that "positive effects of vibration exercise on chronic musculoskeletal pain are evident, and long durations of WBV exercise(s) could be especially beneficial". In addition, research in fibromyalgia patients also found a reduction in pain after 6-weeks of WBV intervention.
Vibration exercise has been shown to improve heart rate variability (HRV) in a range of population, including the elderly. An increase in HRV has been shown to be linked to a reduction in pain, which could benefit patients with IBD.

Limitations to physical activity and pain in IBD cohorts is in part driven by active inflammatory processes, several studies using WBV have demonstrated an increase in anti-inflammatory cytokines in particular IL-10 in various health conditions including fibromyalgia and osteoarthritis. We propose to assess whether this is upheld in Crohn's disease.

Fatigue is a large determinant of HRQoL,as well as a barrier to physical activity as described above. 6 weeks of WBV exercise has been shown to improve fatiguability in different conditions including fibromyalgia and older adults. We know fatigue is multifactorial and includes an aspect of the mental health of the patient, therefore during the study we will ask our patients to undergo a Hospital Anxiety and Depression Scale Questionnaire which is validated in the Inflammatory Bowel Disease Population.

In conclusion: there are several potential mechanisms of action for WBV exercise to improve symptoms and HRQoL of inflammatory bowel disease sufferers. The proposed study would be the first study to explore the concept of using WBV exercise as a potential adjunct therapy in Crohn's disease to improve HRQoL.

2. Aims and Objectives

2.1. Trial/Study objectives

- The primary objective, or principal research question is does WBV exercise improve health related quality of life (HRQoL) for patients with Crohn's disease.

- Secondary Objective: The secondary research questions are:
  - Does WBV improve general cardiovascular health from baseline in Crohn's patients?
  - Is Crohn's disease activity impacted by WBV?
  - Does WBV in Crohn's patients significantly alter their blood markers of inflammation?
  - Does WBV alter bone health markers in CD patients?
  - Does WBV impact on subjective perception of fatigue, or objective measurement of neuromuscular fatigue?
  - Does WBV impact on perception of pain in CD patients?
  - Does WBV impact on markers of anxiety or depression in this population?

-
- Primary End Point: the primary end point is an improvement in the IBD-Q - with an objective improvement of 16 points.

- Secondary End point: The secondary end points are:
  - Improvement in heart rate variability, resting blood pressure or sub maximal Chester step test
  - A static or improved partial Harvey Bradshaw index and faecal calprotectin
  - A significant delta in blood TNF-a, IL-6, IL-17A, IL12/IL-23, IL-10, TGF-beta, C-Reactive protein between the beginning and end of the study
  - An improvement in serum P1NP
  - The difference between subjective fatigue at the start and end of study, as well as a difference in objective neuromuscular fatigue between the start and end of the study
  - Obtaining granularity of patients thoughts, experience and feelings towards WBV as an intervention for the adjunct management of Crohn's disease

3. Methods

3.1. Study Design

VECTOR is a randomised controlled trial.

3.2. Trial/Study Setting

VECTOR recruitment will take place at selected NHS gastroenterology clinics within travelling distance from the University of Hertfordshire Institute of Sport. The baseline, WBV exercise and exit visits will take place in the Institute of Sport, De Havilland Campus, University of Hertfordshire. The lifestyle education for the control arm will take place remotely (on zoom).

3.3. Trial/Study Participants

Adults aged ≥18 years with mild to moderate active CD (Partial Harvey Bradshaw Index of 5-16) will be identified from IBD/Gastro clinics and approached by the clinical team or designated research nurse from 3 NHS sites during the participant gastroenterology outpatient appointment. They will be given the PIS and consent form if interested and asked for contact details so the UH research team can contact them to arrange the initial screening, and subsequent baseline assessment and randomisation visit at the University of Hertfordshire. This information will be emailed from one of the clinical team on their nhs.net account to Dr Jo Brooks-Warburton on Johanne.brooks@nhs.net. Dr Brooks-Warburton will then liaise with the Senior Research Assistant for an appropriate visit time and slot for the participant screening and baseline visit.

Inclusion criteria:

Is the patient aged between 18 and 65 years old (inclusive)?

Has the patient had a clinical diagnosis of Crohn's disease for at least 4 weeks before the screening visit?

Does the patient have either mild or moderate Crohn's disease based on a partial Harvey Bradshaw Index (5-16)?

Does the patient have a faecal calprotectin <250 mcg/g recorded no greater than 4 weeks before the screening visit?

Have the patient's medications been stable (i.e., unchanged) for at least 4 weeks before the screening visit?

Is the patient capable of giving written informed consent?

Is the patient capable of completing the study questionnaires?

Is the patient able to travel to the research sites for all assessment visits and exercise sessions?

Exclusion criteria:

Does the patient have any absolute contraindications to exercise testing and training?

Does the patient have any coexistent serious autoimmune disease?

Does the patient have major surgery planned in the first 3 months following the anticipated baseline visit date?

Is the patient pregnant?

Is the patient female and planning pregnancy within the first 3 months following the anticipated baseline visit date?

Does the patient think that they will be unable to tolerate the venepuncture requirements of the study?

Does the patient lack sufficient venous access to meet the blood sampling requirements of the study?

Is the patient participating in another clinical trial for with concurrent participation is deemed inappropriate?

Is the patient unable to consent?

4. Trial/Study Procedures

4.1. Trial/Study Setup

The trial setup will proceed as follows (further details in GANTT chart below):

1. Obtain ethical approval, set up trial steering group and begin recruiting (Months 1-3)
    a. Sign contracts
    b. Set up trial governance
    c. Collate lifestyle education information from publicly available Crohn's and Colitis UK Patient information.
2. Recruit and enrol 55-60 participants (Months 4-12)
    a. Undertake baseline assessments
    b. Deliver exercise intervention and lifestyle education programme
    c. Undertake post intervention assessments and participant interviews
3. Complete recruitment (12-27 months)
    a. Finish recruiting at 27 months (168 participants in total)
    b. Finish baseline assessments
4. Complete measurements of secondary outcome measures (28-31 months)
    a. Complete interviews
5. Analysis of data, dissemination and report writing (32 – 36 months)
    a. Bloods analysis
    b. Interview analysis
    c. Data analysis
    d. Dissemination – organising workshops to disseminate results

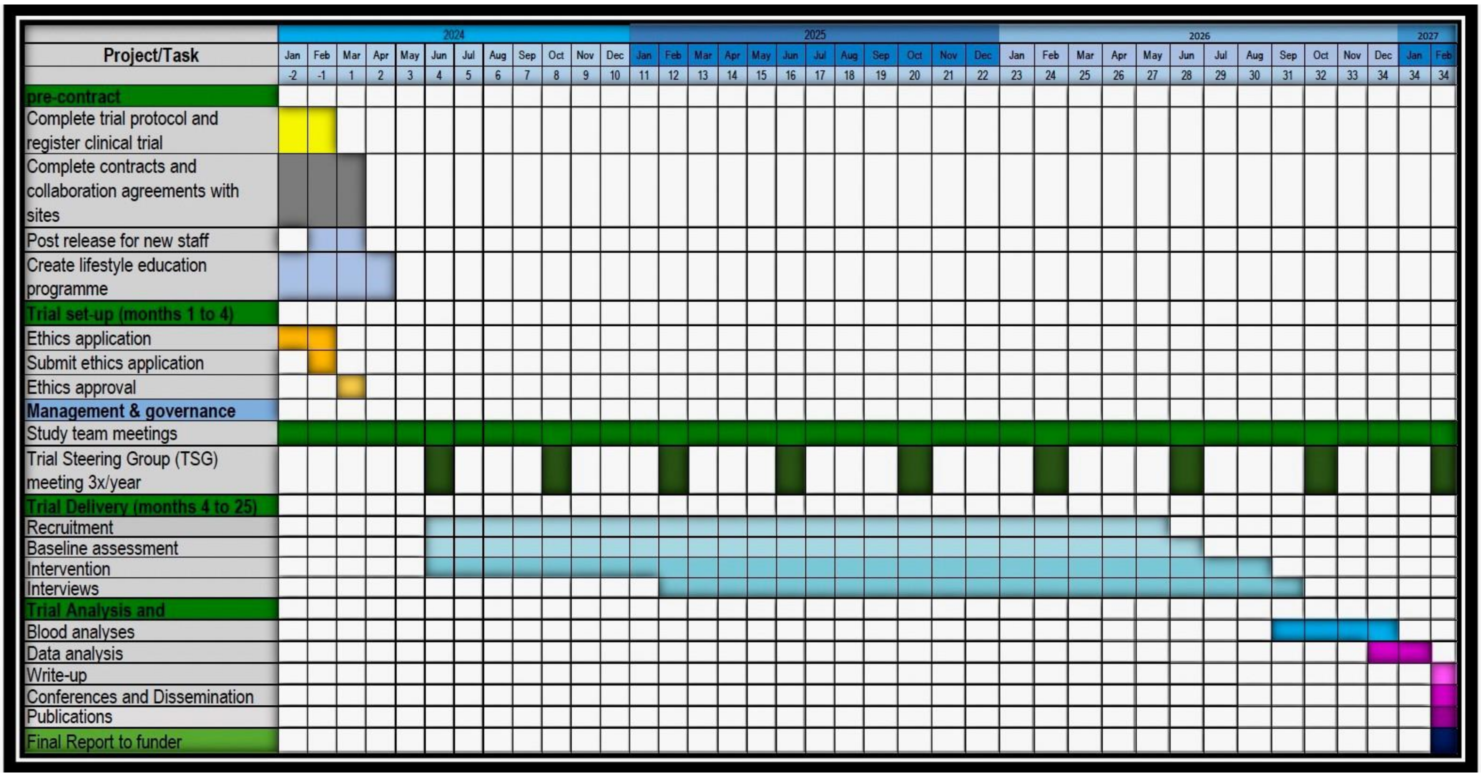

Caption

4.2. Recruitment

4.2.1. Participant Identification

The primary mode of recruitment will be via the IBD clinics at recruiting NHS Hospital Trusts. A member of the clinical team will identify potentially eligible patients via review of medical notes ahead of gastroenterology clinics. Potentially eligible, interested participants will be given the Participant Information Sheet (PIS) to take away and read. Potentially eligible, interested participants will then provide e-consent to share their contact details with the research team using Contact Details Consent Form on the REDCap database. They can then be contacted by a member of the research team within 28 days to organise the screening, trial consent and randomisation visit.

4.3. Screening

Potentially eligible patients will be initially screened for participation in the study by a research nurse with eligibility confirmed with a delegated clinical co-investigator. Once e-consent to share participant contact details has been gained, the baseline/screening visit must be performed with 28 days with the research team. During this screening ALL participants who are biologically able to become pregnant will be asked to complete a pregnancy test as part of the eligibility criteria, as pregnancy or planned pregnancy within 3 months of enrolling in the study is an exclusion criteria.

4.3.1. Informed Consent

There are two stages of informed consent. As stated above, participants are e-consented to share their contact details with the research team using Contact details consent form on the REDCap database. They are reminded of the attributes of the study at this stage.

Following screening, informed e-consent to take part in the study is gained via the VECTOR consent form on REDCap, with the research team. It is the responsibility of the Investigator, or appropriately trained person delegated by the Investigator as documented in the site delegation log, to obtain informed consent from each participant prior to any participation or trial/study specific procedures. This should follow adequate explanation of the aims, methods, anticipated benefits and potential hazards of the trial/study.

The Investigator (or other qualified person on the delegation log) will explain to the potential participant that they are free to refuse any involvement within the trial/study or alternatively withdraw their consent at any point during the trial/study and for any reason.

If there is any further safety information which may result in significant changes in the risk/ benefit analysis, the PIS and Vector Consent Form will be reviewed and updated accordingly. All participants that are actively enrolled on the trial/study will be informed of the updated information and given a revised copy of the PIS in order to confirm their wish to continue on the trial/study.

Further guidance can be found at: http://www.hra.nhs.uk/research-community/before-you-apply/participant-information-sheets-and-informed-consent/

4.4. Allocation/Randomisation

4.4.1. Randomisation Procedures

Once screening procedures have confirmed the participant's eligibility to enter the trial/study and the participant has provided informed consent, the process of randomisation can commence.

The participants will be 1:1 randomised using REDCAP (an automated process) after screening confirmation and consent, this process is immediate and will be done whilst the participant is undergoing baseline measurements (as defined below). This is done by the member of the research team undertaking the screening and consent (e.g. the research assistant). As the trial cannot be blinded there is no code break.
Once the participant has been successfully randomised in VECTOR, the enrolment of this participant must be documented within an enrolment log.

4.5. Baseline

Baseline tests are:
1. Submaximal Chester Step Test
2. Anthropometry: Weight, Height, heart rate variability, blood pressure
3. *Bloods for inflammatory markers as described above
4. Electromyography (EMG)
5. Health Related Quality of Life Questionnaire (IBD-Q)
6. HADS questionnaire
7. IBD-Fatigue Questionnaire
8. Faecal calprotectin (done as part of usual care at the NHS site, and only the result is used, no stool samples will be handled by the research team)
9. REDCAP participant 7 day input of pain perception and physical activity.

*12ml of whole blood will be taken via venipuncture, by a team member training in phlebotomy who is on the delegation log. The blood will be centrifuged on site, the cellular component destroyed and discarded as per Health and Safety guidelines, the remaining acellular plasma will be stored in vials labelled with the participant pseudoanonymised number at -80 degrees centigrade, and then once all samples are collected from the study, they will be sent to Dr Lawson's Laboratory at The University of Central Lancashire by courier.

4.6. Intervention(s)

The whole body vibration arm participants will have 18 visits to the University of Hertfordshire for their intervention (20 visits in total (+1 if they engage with the semi structured interviews). The WBV will involve performing static squatting on a synchronic vibrating platform. Patients will hold a squatting position with 30˚ of knee flexion, with their feet about 30cm apart, barefoot and with upper limbs holding the platform bars, for 60 seconds, then rest for 60 seconds, and repeat this a total of six times. The vibratory stimulus will be offered at an amplitude of 1.5mm with a frequency of 30 Hz.

During their visit, they will have 10 minutes of the lifestyle education programme, totaling 30 minutes per week.

Lifestyle education programme (30 minutes total):
Week 1 - Healthy Eating Information from Crohn's and Colitis UK leaflets
Week 2 - Discussing barriers and how to overcome them to Healthy Eating
Week 3 - Physical Activity for IBD (Crohn's and Colitis video)
Week 4 - Barriers to Physical Activity
Week 5 - Overcoming barriers and positive steps forward to engage in Physical Activity.
Week 6 - Smoking Cessation if applicable, round up of the last 5 weeks.

For the comparator arm - this programme will be delivered individually via zoom in one 30 minute session per week.

4.7. Follow up Procedures

Any participants who exit the study prior to completion will be invited to feedback regarding the study.
The closing visit (if not undertaking the semi structured interviews) for the participant, will be a repeat of the baseline visit held at the University of Hertfordshire;
1. Submaximal Chester Step Test
2. Anthropometry: Weight, Height, heart rate variability, blood pressure
3. *Bloods for inflammatory markers as described above
4. Electromyography (EMG)
5. Health Related Quality of Life Questionnaire (IBD-Q)
6. HADS questionnaire
7. IBD-Fatigue Questionnaire
8. Faecal calprotectin result (done at the NHS site where their Gastroenterology Consultant is, and only the result is used, no stool samples will be handled by the research team)
9. REDCAP participant 7-day input of pain perception and physical activity.

The first 15 participants to complete the intervention arm, who agree, will be invited to 1:1 semi-structured interview regarding the WBV exercise to gain more granularity regarding the experience, thoughts and feelings regarding WBV as an adjunct therapy for Crohn's disease.

*12ml of whole blood will be taken via venipuncture, by a team member training in phlebotomy who is on the delegation log. The blood will be centrifuged on site, the cellular component destroyed and discarded as per Health and Safety guidelines, the remaining acellular plasma will be stored in vials labelled with the participant pseudoanonymised number at -80 degrees centigrade, and then once all samples are collected from the study, they will be sent to Dr Lawson's Laboratory at The University of Central Lancashire by courier.

4.8. End of Study

The End of the study is when all participants have completed all scheduled assessments and all questionnaire/data items have been completed and for those undertaking it, the semi-structured interview has been undertaken.

5. Safety

Adverse event reporting will be conducted in accordance with the Sponsor"s Adverse Event Reporting Procedures. A clinical co-investigator will be responsible for determining the causality and seriousness of adverse events, as well as all non-serious adverse events that are either deemed to be related to participation in the research or result in withdrawal from the WBV programme or study.

Serious adverse events are defined as any untoward medical occurrence that results in one of the following criteria: results in death; is life threatening; requires unplanned or prolonged hospitalization; results in persistent or significant disability or incapacity, or; results in a congenital abnormality or birth defect. Non-serious events are defined as any untoward medical occurrence that does not fulfil any of the serious adverse event criteria.

6. Data Collection, Management and Analysis

6.1. Data Collection

Data to be collected (*pre intervention and post intervention, +data taking once, then using pseudoanonymised number in the database):
- +Name
- +Contact details
- +Age
- +Gender

- *Weight
- *Height
- *Resting blood pressure
- *Heart rate variability
- *Harvey Bradshaw Index (partial)
- *EMG
- *Blood TNFalpha, IL10, IL12, IL23, TGF-beta, IL6, IL17, CRP, P1NP
- *Faecal calprotectin x 2 via their gastroenterologist
- *HADs
- *IBD-Q
- *IBD-F
- *7 day pain perception
- *7 day level of physical activity engagement
- *Cardiorespiratory fitness (sub maximal Chester Step Test)

6.2. Data Management

Essential Trial documentation (i.e. the documents which individually and collectively permit evaluation of the conduct of a clinical trial and the quality of the data produced) will be kept with the Trial Master File and Investigator Site Files. The Sponsor will ensure that this documentation will be retained for a minimum of five years after the conclusion of the trial to comply with standards of Good Clinical Practice. Case report forms will be stored for a minimum of five years after the conclusion of the trial as paper records, and a minimum of 20 years in electronic format. All paper records will be stored in a secure storage facility or off-site by the University of Hertfordshire. All electronic records will be stored on a password protected server.

6.3. Data Analysis

All analyses of the intervention-based data will be performed on an intention to treat basis.

To determine the effects of the intervention on all of the outcome measures, differences in the changes from baseline to after the 6 week intervention between the two groups will be examined using linear mixed effects models with group modelled as a fixed factor and random intercepts by participants adopted. For linear mixed models the mean difference between groups in change from baseline to after the 6 week intervention and 95% confidence intervals of the difference will be presented.
Effect sizes and their associated 95% confidence intervals will also be calculated for the changes from baseline to after the 6 weeks of intervention between the two groups, using Cohen"s d, in accordance with McGough, & Faraone. Cohen"s d values will be interpreted as 0.2 = small, 0.5 = medium, and 0.8 = large .

Analyses will be conducted in SPSS (IBM, SPSS, V28). The flow of participants through each stage of the trial will be presented in a CONSORT diagram. Descriptive statistics of means and standard deviations will be used to characterise the groups at baseline and differences between the two groups
will be presented in the form of an unadjusted mean difference for continuous outcomes, with their associated 95% confidence intervals.
In addition, Pearson chi-square tests of independence will be utilized to undertake bivariate crosstabulation comparisons between the two trial groups to test differences in the number lost to follow-up and the number of adverse outcomes in each group. Chi-squared probability values will be calculated using Monte Carlo simulation. All statistical analyses will be conducted using SPSS v28 (IBM, SPSS), with statistical significance accepted at the $P \leq 0.05$ level.

7. Ethical Considerations

The study will be undertaken according to the principles of ICH Good Clinical Practice (GCP), and all relevant ethics and governance processes, including the HRA approvals. All of the research team will be GCP compliant.

Before the study starts, REC approval via IRAS, HRA approval and University of Hertfordshire local ethics review approval will be sought. NHS Research and Development team at the East and North Hertfordshire Trust, Guys and St Thomas' NHS Trust and Luton and Dunstable NHS Trust will need to review and accept the study. NIHR Portfolio status will be sought.

The CI or PI at the lead site (ENHT) will complete an annual progress report to be submitted to the REC and each NHS R&D department.

8. Quality Assurance and Control

8.2. Risk Assessment

This study is determined as low risk; the rights and safety of participants will be upheld throughout. All participants will perform a warm up and cool down for every WBV session, however muscle soreness has been identified as a risk factor. WBV is low intensity and undertaken with 1:1 supervision, which with the warm/cool down reduces this risk. The research assistant will be first aid trained, and there is a defibrillator on site. All risks associated with the study have been assessed using the University of Hertfordshire's Life and Medical Sciences (LMS) risk assessment form.

The project concept is being supported by an established and experienced Trial Management Group and the Lead PI is being supported and mentored to run the trial via the CI who has extensive experience in this style of research.

The Trial is supported by the UH CTSN in terms of QC and QA to ensure the study is performed and data generated/documented and reported in compliance with the principles of GCP, they are also reportable to regarding study recruitment rates.

8.3. Monitoring

The site PIs will be responsibility for reviewing participant medical data, the CI, Lead PI and Research Assistant with be responsible for reviewing data errors and missing data on the database. Site visits will occur annually by the CI/delegated responsible person. As the participants will be attending the University of Hertfordshire site, the Lead PI and Research Assistant will be monitoring the study at site level. The Study will be subject to monitoring audits as per the University of Hertfordshire CTSN.

8.4. Study Oversight

8.4.3. Trial Management Group

The study is overseen by the Trials Management Group, consisting of the CI (and Exercise Science Specialist), the Lead PI (Gastroenterologist and Researcher), the Senior Research Assistant, the Statistician, Patient Participant, Immunologist, Sports Psychologist (qualitative research specialist), PI/Gastroenterologist and Bio-Engineer. They meet quarterly to input into the design and delivery of the trial, ensure recruitment is progressing and ensure the study is running to target and after the study period support the dissemination programme of the results. The CTSN will also have oversight of the study.

9. Public and Patient Involvement

PPI has been integral to the design of the study and each of the patient facing documents have had input from the Patient Participant on the TMG. The CI has lived experience of Crohn's so brings this aspect also to the study. 86% of patient volunteers who undertook a survey regarding whole body vibration exercise state they would be willing to take part in a study regarding this. Tolerability has also been assessed with a pilot study of WBV in Crohn's volunteers. These have moulded the current study design.

10. Protocol Compliance

The CI will ensure that the trial is conducted in compliance with the principles of the Declaration of Helsinki (1996), and in accordance with all applicable regulatory requirements including but not limited to the Research Governance Framework, Trust and Research Office policies and procedures and any subsequent amendments.

The sponsor will maintain a log of the non-compliances to ascertain if there are any trends developing which to be escalated. The sponsor will assess the non-compliances and action a timeframe in which they need to be dealt with. Each action will be given a different timeframe dependent on the severity. If the actions are not dealt with accordingly, the R&D Office will agree an appropriate action, including an on-site audit.

11. Data Protection and Participant Confidentiality

Confidentiality
The Investigator has a responsibility to ensure that participant anonymity is protected and maintained. They must also ensure that their identities are protected from any unauthorised parties. Information with regards to study participants will be kept confidential and managed in accordance with the Data Protection Act, NHS Caldicott Guardian, The Research Governance Framework for Health and Social Care and Research Ethics Committee Approval.

Patient identifiable information shared across the research team is limited to name, email address and phone number. Hospital number and date of birth is collected on CRFs at the NHS site - but pseudo-anonymised with a patient research number. Only members of the research team will have access to this information.
The CI is custodian of the data.

The participants can withdraw at any stage, but any data collected up until the point f the withdrawal will still be used within the study. The patients will be asked for a preference of contact details and can withdraw this at anytime by informing the research team.

All data will be anonymised within any publications or dissemination of findings. The CI is the data controller and the research teams both on NHS site and at the University of Hertfordshire are the data handlers. The responsibility for data quality is described above. DPIAs will be completed for each NHS site with the University of Hertfordshire.

Case Report Form (CRF) forms are visit specific and are completed by the research assistant or member of the research team undertaking that particular visit parameter.

The Baseline and exit CRFs will record the data as described above
The WBV CRFs will confirm the number of WBV 60 seconds undertaken and confirmation of the lifestyle education delivery.
It will also confirm any AEs during the WBV intervention.

Record Retention and Archiving

During the course of research, all records are the responsibility of the CI and are kept in secure conditions. The completed paper forms with non participant identifiable data are kept with the CI in a locked filing cabinet behind ID card security doors. Medical information on CRFs are kept in locked cabinets in the Research Department in the NHS Trusts. The records are retained on a secure repository at the University of Hertfordshire. Any electronic data is kept in encrypted storage which is password protected. When the research trial is complete, records are kept for a minimum of 5 years as per standards of GCP.

Compliance
The CI will ensure that the trial is conducted in compliance with the principles of the Declaration of Helsinki (1996), and in accordance with all applicable regulatory requirements including but not limited to the Research Governance Framework, Trust and Research Office policies and procedures and any subsequent amendments.

12. Publication and Dissemination

The findings will be written up in journal publications and evidence briefings and presented at various forums and conferences. Findings will importantly be reported back to patients across the UK via networks such as Crohn"s and Colitis Foundation, healthcare professionals and a wider audience.
